# Clinical analysis of transurethral holmium laser enucleation in the treatment of benign prostatic hyperplasia with prostatic inflammation: A prospective research study

**DOI:** 10.3389/fsurg.2022.1026657

**Published:** 2023-01-06

**Authors:** Weijian Zhou, Dongdong Mao, Liang Li, Gang Liu, Guojun Gao, Haikun Li, Dianjun Gao

**Affiliations:** ^1^ Department of Clinical Medicine, Weifang Medical University, Weifang, China; ^2^ Department of Urology, Affiliated Hospital of Weifang Medical University, Weifang, China

**Keywords:** benign prostatic hyperplasia, prostatic inflammation, HoLEP, clinical efficacy, prospective research

## Abstract

**Objective:**

To investigate the clinical efficacy of holmium laser enucleation of the prostate (HoLEP) in the treatment of benign prostatic hyperplasia (BPH) with prostatic inflammation (PI).

**Methods:**

We prospectively collected and followed up data on patients with BPH who underwent HoLEP at the Affiliated Hospital of Weifang Medical University between July 2021 and July 2022. According to the postoperative pathological results, the patients were divided into two groups: BPH without PI group (BPH group) and BPH with PI group. Statistical analysis was performed on clinical data, including age and body mass index (BMI), prostate volume (PV), postoperative residual urine volume (PVR), preoperative serum total prostate-specific antigen (tPSA), serum-free prostate-specific antigen (fPSA), preoperative and postoperative maximum urinary flow rate (Qmax), International Prostate Symptom Score (IPSS) before and 3 months after surgery, quality of life index (QoL) before and 3 months after surgery, and postoperative complications.

**Results:**

A total of 41 patients were included in this study, including 16 in the BPH group and 25 in the BPH with PI group. There were no significant differences in preoperative age, BMI, PV, PVR, tPSA, fPSA, and f/tPSA between the BPH and BPH with PI groups (*P* > 0.05). The preoperative mean Qmax of the BPH and BPH with PI groups were 9.44 ± 2.449 and 7.52 ± 2.946 [mean ± standard deviation (SD)] ml/s, mean IPSS were 17.75 ± 5.335 and 24.24 ± 5.861 (mean ± SD), and mean QoL were 4.13 ± 0.806 and 4.48 ± 0.8 (mean ± SD), respectively. The postoperative mean Qmax of the BPH and BPH with PI groups were 20.38 ± 4.787 and 14.32 ± 3.827 (mean ± SD) ml/s, mean IPSS were 2.69 ± 1.25 and 5.84 ± 3.579 (mean ± SD), and mean QoL were 0.13 ± 0.342 and 0.92 ± 0.759 (mean ± SD), respectively. In both groups, Qmax significantly increased (*P* < 0.05) and IPSS and QoL significantly decreased after HoLEP (*P* < 0.05). Before and after surgery, the Qmax in the BPH with PI group was lower than that in the BPH group, and the IPSS and QoL levels in the BPH with PI group were higher than those in the BPH group (*P* < 0.05). Compared with the BPH group, the increase in Qmax in the BPH with PI group was smaller and the decrease in IPSS was larger (*P* < 0.05), but the variation in QoL was not statistically significant (*P* > 0.05).

**Conclusion:**

Improvements in Qmax, IPSS, and QoL in BPH patients with PI after HoLEP surgery were lower than those in BPH patients alone. PI may be a predictor of a worse response to surgical treatment. However, more multicenter randomized controlled trials with larger samples and long-term follow-up are needed to verify this.

## Introduction

Benign prostatic hyperplasia (BPH) is one of the most common diseases in older men, with a prevalence of 30% in those over the age of 50 years (1). Prostatic inflammation (PI) is also a common genitourinary disease in men, with a prevalence of 12.4% for prostatitis-like symptoms in the total population (2). The prevalence rate among young men aged 18–30 years is as high as 16% (3). However, a combination of these two diseases is present in approximately 5%–20% of cases (4). One study reported that inflammatory cell infiltration was found in 81% of postoperative pathological tissues in patients with BPH (5). Moreover, prostate inflammation plays a major role in BPH development and pathogenesis (6, 7). In recent years, with advancements in minimally invasive techniques, holmium laser enucleation of the prostate (HoLEP) has been applied effectively in clinical practice. Compared with traditional minimally invasive methods, such as transurethral plasma enucleation and transurethral resection of the prostate, HoLEP shows superior clinical efficacy and safety in the treatment of patients with BPH (8). In this study, we collected data from patients who underwent HoLEP at our hospital. We aimed to explore the clinical characteristics of BPH complicated with PI before and after surgery and provide new ideas to improve the clinical outcomes of these patients.

## Materials and methods

### Subjects

The study was approved by the Medical Research Ethics Review of the Affiliated Hospital of Weifang Medical University (approval number: wyfy-2022-ky-172). Using a prospective design, we included patients who met the following criteria: (i) patients who underwent HoLEP at the Affiliated Hospital of Weifang Medical University from July 2021 to July 2022 and (ii) patients with or without PI, as long as the postoperative pathological findings confirmed BPH. PI was defined as pathological inflammation of the prostate specimen after resection, including postoperative pathology suggestive of chronic or acute prostatitis. We excluded patients with a previous history of prostatic surgery and/or biopsy, acute urinary retention, urinary tumors, severe metabolic diseases, multiple organ dysfunction and other diseases, or incomplete clinical data.

### Grouping

According to the postoperative pathological results, the patients were divided into two groups: BPH without PI group (BPH group) and BPH with PI group.

### Methods

The following parameters were obtained and recorded: (i) patient age and body mass index (BMI); (ii) prostate volume (PV) and postvoid residual urine (PVR): PV and PVR determined by urological ultrasound = anterior-posterior diameter × superior-inferior diameter × left-right diameter × 0.523; (iii) preoperative prostate-specific antigen (PSA): fasting serum PSA in the morning was determined by enzyme-linked immunosorbent assay (ELISA), including total PSA (tPSA) and free PSA (fPSA); (iv) maximum flow rate (Qmax) before surgery and 1 day after catheter removal post-surgery: measured using Flowmaster wireless Uroflowmeter (Laborie Medical Technologies, USA); (v) International prostate symptom score (IPSS) before and 3 months after surgery, where the higher score, the more severe the lower urinary tract symptoms (LUTS); (vi) quality of life (QoL) before and 3 months after surgery, where the higher the score, the more distressed the patients are.

### Statistical analysis

THE IBM SPSS Statistics for Windows, version 26.0 (IBM Corp., Armonk, N.Y., USA) was used for statistical analysis. We used the Shapiro-Wilk test to verify the normality of the data. The *F*-test was used to determine the homogeneity of variances. Normally distributed continuous variables are expressed as mean ± standard deviation and compared using *t*-tests. Two independent sample *t*-tests were used for comparisons between the groups, and a paired *t*-test was used for comparisons before and after surgery. Statistical significance was set at *P* < 0.05.

## Results

### Comparison of clinical indicators between the two groups before surgery

A total of 41 patients were included in this study: 16 in the BPH group and 25 in the BPH with PI group. In this study, there were no significant differences in preoperative age, BMI, PV, PVR, tPSA, fPSA, and f/tPSA between the two groups (Table 1).

**Table 1:** Comparison of clinical indicators between two groups of patients before surgery (mean ± SD).

Clinical indicators	BPH (*n* = 16)	BPH with PI (*n* = 25)	*t*	*P*-value
Age (years)	65.81 ± 2.79	67.36 ± 4.15	−1.311	0.198
BMI	24.74 ± 2.51	23.68 ± 2.46	1.338	0.189
PV (ml)	47.34 ± 17.09	55.17 ± 35.67	−0.817	0.419
PVR (ml)	55.74 ± 91.48	60.73 ± 100.35	−0.161	0.873
tPSA (ng/ml)	3.93 ± 2.29	4.12 ± 3.50	−0.2	0.842
fPSA (ng/ml)	0.84 ± 0.46	0.85 ± 0.67	−0.058	0.954
f/tPSA	0.24 ± 0.09	0.28 ± 0.17	−0.8	0.429

BPH, benign prostatic hyperplasia; PI, prostatic inflammation; BMI, body mass index; PV, prostate volume; PVR, postvoid residual urine; tPSA, total prostate-specific antigen; fPSA, free prostate-specific antigen; f/tPSA, fPSA/tPSA; SD, standard deviation.

### Comparison of Qmax data between the two groups before and after surgery

Preoperative mean Qmax of the BPH and BPH with PI groups were 9.44 ± 2.449 and 7.52 ± 2.946 [mean ± standard deviation (SD)] ml/s, and postoperative mean Qmax were 20.38 ± 4.787 and 14.32 ± 3.827 (mean ± SD) ml/s, respectively. The results of the intra-group comparison showed that the postoperative Qmax in the BPH with PI and BPH groups was significantly higher than the preoperative Qmax in the BPH with PI and BPH groups (*P* < 0.001). Moreover, compared with the BPH group, the variation in Qmax values in the BPH with PI group was lower in the preoperative, postoperative, and pre- and postoperative Qmax, and all differences were statistically significant (*P* < 0.05) (Table 2).

**Table 2:** Comparison of Qmax data between two groups before and after surgery (mean ± SD).

Indicators	Sample size	Qmax (ml/s)				
		Pre-operation	Post-operation	Variation	*t*	*P*-value
BPH	16	9.44 ± 2.449	20.38 ± 4.787	−10.94 ± 3.17	−13.792	<0.001
BPH with PI	25	7.52 ± 2.946	14.32 ± 3.827	−6.8 ± 3.873	−8.779	<0.001
*t*		2.166	4.48	3.57		
*P*-value		0.036	<0.001	0.001		

BPH, benign prostatic hyperplasia; PI, prostatic inflammation; Qmax, maximum flow rate; SD, standard deviation.

### Comparison of IPSS data between the two groups before and after surgery

Preoperative mean IPSS of the BPH and BPH with PI groups were 17.75 ± 5.335 and 24.24 ± 5.861 (mean ± SD), and postoperative mean IPSS were 2.69 ± 1.25 and 5.84 ± 3.579 (mean ± SD), respectively. The results of the intra-group comparison showed that the postoperative IPSS of the BPH with PI and BPH groups were significantly lower than those before surgery, and the differences before and after surgery were statistically significant (*P* < 0.001). Moreover, the IPSS of the BPH with PI group was higher than that of the BPH group before and after surgery, and the difference between the two groups was statistically significant (*P* < 0.05). The variation in the IPSS before and after surgery in the BPH with PI group was larger than that in the BPH group, and the difference between the two groups was statistically significant (*P* < 0.05) (Table 3).

**Table 3:** Comparison of IPSS data between two groups before and after surgery (mean ± SD).

Indicators	Sample size	IPSS				
		Pre-operation	Post-operation	Variation	*t*	*P*-value
BPH	16	17.75 ± 5.335	2.69 ± 1.25	15.06 ± 4.611	13.066	<0.001
BPH with PI	25	24.24 ± 5.861	5.84 ± 3.579	18.4 ± 5.26	17.491	<0.001
*t*		−3.578	−4.037	2.076		
*P*-value		0.001	<0.001	0.044		

BPH, benign prostatic hyperplasia; PI, prostatic inflammation; IPSS, international prostatic symptom score; SD, standard deviation.

### Comparison of QoL data between the two groups before and after surgery

The preoperative mean QoL of the BPH and BPH with PI groups were 4.13 ± 0.806 and 4.48 ± 0.8 (mean ± SD), and the postoperative mean QoL was 0.13 ± 0.342 and 0.92 ± 0.759 (mean ± SD), respectively. The results of the intra-group comparison showed that the postoperative QoL in the BPH with PI and BPH groups were significantly lower than those before surgery, and the differences were statistically significant (*P* < 0.001). Furthermore, QoL in the BPH with PI group was higher than that in the BPH group before and after surgery, and the difference between the two groups was statistically significant (*P* < 0.05). There was no significant difference in the variation in QoL between the two groups before and after surgery (Table 4).

**Table 4:** Comparison of QoL data between two groups before and after surgery (mean ± SD).

Indicators	Sample size	QoL				
		Pre-operation	Post-operation	Variation	*t*	*P*-value
BPH	16	4.13 ± 0.806	0.13 ± 0.342	−4 ± 0.816	19.596	<0.001
BPH with PI	25	4.48 ± 0.8	0.92 ± 0.759	−3.92 ± 1.152	17.017	<0.001
*t*		−2.783	−3.927	−0.241		
*P*-value		0.008	<0.001	0.811		

BPH, benign prostatic hyperplasia; PI, prostatic inflammation; QoL, quality of life; SD, standard deviation.

## Discussion

BPH and PI are common urinary diseases in middle-aged and older men and mainly affect their QoL (9, 10). Tang et al. found that the incidence of BPH complicated with PI was as high as 78.3% (11). A multicenter study by Nickel et al. showed that 77.6% of 8,224 patients with BPH had PI (12). In a study by Cao et al., 91.7% of the patients with BPH had PI (13). The BPH rate of combined PI was as high as 78.6% in a study by Li et al. (14). However, in a study by Chang et al., only 37.5% of pathological specimens from patients with BPH had PI (15). Similarly, only 34.2% of the patients with BPH in the study by Du et al. had PI (16). In contrast, the percentage of patients with BPH and PI in the present study was 61.0%. Such large differences may be related to the different pathological specimen collections and pathological diagnostic criteria.

The present study showed no statistically significant difference in PV between the BPH with PI and BPH groups (*P* > 0.05). However, Long et al. showed that the PV of BPH patients with PI was significantly higher than that of BPH patients without PI (17). Gerstenbluth et al. also showed that patients with BPH who had PI had significantly larger PV than those with BPH only (18). Inflammation of the prostate leads to repeated destruction, healing, and regeneration of prostate tissue, causing enlargement and remodeling of prostate nodules (19). The difference in the results of the current study may be related to the insufficient sample size or the lower degree of inflammation found in the included patients. There were no statistical differences between the two groups in the present study in the indicators of PVR, tPSA, fPSA, and f/tPSA., which is the same as the results of Du et al. and Li et al. (14, 16). However, Feng et al. found that tPSA was significantly higher in the BPH with PI group than in the BPH group (20). Most of the current studies suggest that inflammatory cells in PI infiltrate the prostate epithelium, causing its destruction and release of excessive PSA, and that the prostatic ducts and the original physiological barrier in BPH patients with PI are severely disrupted by inflammation and PSA leaks into the circulation, leading to elevated serum PSA levels (21–23). PI may be an important factor influencing the elevation of PSA levels; however, its significance needs to be supported by more data.

The present study focused on evaluating the clinical outcomes of two groups of patients undergoing HoLEP using two subjective indicators (IPSS and QoL) and one objective indicator (Qmax). The American Urological Association recommends the former to evaluate the severity of LUTS and is the best method for evaluating the severity of symptoms in patients with BPH (24). The QoL score is mainly used to evaluate the degree of LUTS distress and tolerance in patients with BPH (25). Qmax can be used as an indicator to assess disease progression and the preoperative and postoperative surgical outcomes in patients with BPH. Our study found that the Qmax in the BPH with PI group was lower than that in the BPH group, whereas the IPSS and QoL in the inflammation group were higher than those in the BPH group before and after surgery (*P* < 0.05). It has been suggested that inflammation may aggravate the clinical symptoms of patients with BPH, especially LUTS. Robert et al. and the REDUCE study similarly showed a higher preoperative IPSS in BPH patients with inflammation than in patients with BPH alone (5, 12). A study by Du et al. also showed that, both preoperatively and postoperatively, the group with inflammatory hyperplasia had lower Qmax and higher IPSS and QoL than the group with simple hyperplasia (16). Although that study was based on patients with BPH after plasma resection, similar results confirm that PI promotes disease progression and affects the QoL of patients. Related studies have shown that PI can induce T-cell activation under certain initial stimulations, thus producing and releasing related inflammatory factors (such as IL-6 and IL-8) that lead to cell injury (26). Related inflammatory factors can also react with prostatic stromal and other cells to stimulate prostate tissue hyperplasia (27). The processes of lymphocyte activation, cytokine release, and tissue hyperplasia can act as a self-perpetuating cycle, leading to chronic inflammation and a gradual increase in the volume of the prostate, thus forming a “vicious circle” (28).

Furthermore, the present study showed that compared with the BPH group, the increase in Qmax was smaller and the decrease in IPSS was larger in the group with inflammation, but there was no significant difference in the variation in QoL. In contrast, a study by Huang et al. showed no statistically significant difference in IPSS variation after transurethral resection of the prostate (TURP) in patients with BPH with or without PI (29). Can we speculate that HoLEP surgery can reduce IPSS to a greater extent than TURP in patients with BPH with PI, that is, are HoLEP surgeries more effective in relieving LUTS? The mechanism of HoLEP treatment, that is, vaporization, rapid coagulation, and thin-layer engagement, has this basis (30). Further research is required to confirm this hypothesis.

The present study had some limitations. First, the sample size of the present study was relatively small, and the sample was limited to a single medical center. Second, our research did not investigate or restrict patients’ pre-surgical medications, such as whether they were taking antibiotic-like or antispasmodic drugs. This may have influenced the results. Furthermore, the same surgeon did not complete each patient's operation, which may have affected the outcome to a certain extent. The follow-up period of this study was short, and the results obtained were limited and one-sided. We lacked long-term relevant data to confirm the reliability of the present study. However, the sample in this study was strictly screened and the results were relatively robust, which can still serve as a guide for clinical work.

In conclusion, improvements in Qmax, IPSS, and QoL in BPH patients with PI undergoing HoLEP were lower than those in patients with BPH alone. PI may be a predictor of a worse response to surgical treatment. However, the next step is to expand the sample size to a multicenter study and conduct a comparative study on the safety and clinical efficacy of different clinical interventions in BPH patients with PI undergoing HoLEP.

## Data Availability

The original contributions presented in the study are included in the article/Supplementary Material, further inquiries can be directed to the corresponding author/s.
